# Incident reporting among physicians‐in‐training in Japan: A national survey

**DOI:** 10.1002/jgf2.454

**Published:** 2021-05-25

**Authors:** Masaru Kurihara, Yoshimasa Nagao, Yasuharu Tokuda

**Affiliations:** ^1^ Department of Hospital Medicine Urasoe General Hospital Okinawa Japan; ^2^ Department of Patient Safety Nagoya University Hospital Nagoya Japan; ^3^ Muribushi Okinawa Center for Teaching Hospitals Okinawa Japan

**Keywords:** incident reporting, medical education, patient safety

## Abstract

**Background:**

Incident reporting can inform hospital safety. However, under‐reporting is preventing this.

**Methods:**

We conducted a nationwide survey among Japanese physicians‐in‐training by including a questionnaire in the General Medicine In‐Training Examination to assess incident reporting behavior and participation in patient safety lectures.

**Results:**

Responses of 6,164 physicians‐in‐training indicated that although 78% had attended patient safety lectures, 44% had not submitted an incident report in the previous year and 40.6% did not know how to submit an incident report.

**Conclusions:**

The discrepancy between attendance at safety courses and incident reporting behavior must be addressed to improve hospital safety.

## 1 BACKGROUND AND INTRODUCTION

Incident reporting systems in hospitals are used for identifying and recording adverse events.^1^ By analyzing these reports, knowledge of the characteristics and causes of incidents is obtained, and measures are taken to prevent their recurrence. More than 90% of Japanese hospitals use an established incident reporting system, and an association has been found between the reporting of incidents and the improvement of safety culture.^2^ Under‐reporting of incidents is recognized as a significant problem in some countries including Japan.^3^, ^4^

Residents, or physicians‐in‐training, are frontline healthcare providers in teaching hospitals. In Japan, after graduating from medical school, all physicians must spend the first two years of their careers as a physician‐in‐training. Previous reports have indicated that increasing resident reporting may reveal systemic issues not previously identified, which, if addressed, could improve patient safety.^5^ However, the extent of residents’ actual reporting behavior has been unclear. The current study is the first nationwide survey of physicians‐in‐training in Japan to evaluate the actual extent of incident reporting.

## 2 MATERIALS AND METHODS

A questionnaire was included in the General Medicine In‐Training Examination (GM‐ITE). This multiple‐choice examination was developed by the committee of Japan Institute for Advancement of Medical Education Program for the same purpose as the Internal Medicine In‐Training Examination in the United States (IM‐ITE): to determine the performance of each resident and each program.^6^ The questionnaire concerned patient safety (see Table 1). The program directors of each hospital administered the questionnaires with the examination in January 2020. Only those residents or physicians‐in‐training who agreed and provided informed consent answered the questionnaire. The program directors emailed the anonymized responses to the researchers. This study was approved by the institutional review board. Statistical analysis was performed using STATA version 11 (Stata Corporation, College Station, TX, USA). *p* < 0.05 was considered to be statistically significant.

**TABLE 1:** Questionnaire about patient safety

Q1: How many incidents are reported per year during the training?
Q2: Do you know how to complete an incident report in your hospital?
Q3: Have you ever participated in a lecture on patient safety?

## 3 RESULTS

A total of 6,869 residents (539 hospitals) participated in the GM‐ITE. Of these, we analyzed the responses of 6,164 physicians‐in‐training who volunteered to participate in the survey (response rate 89.7%). The participants comprised 594 women (9.6%) and 5,570 men (90.4%). There were 3,412 first‐year residents (55.4%) and 2,752 second‐year residents (44.6%). Of the participants, 5,376 residents (78％; 95% CI, 0.77−0.79) reported that they had participated in a lecture on patient safety in their hospitals. However, 2,735 reported that they had not submitted an incident report in the previous year (44％; 95% confidence interval (CI), 0.43−0.45) and 2,512 residents (37%; 95% CI, 0.35−0.38) reported that they had submitted 1−5 incident reports during the previous year. A total of 103 residents submitted an incident report without participating in a lecture on patient safety. Additionally, 3,864 of them reported that they did not know how to submit an incident report (56％; 95% CI, 0.55−0.57).

## 4 DISCUSSION AND CONCLUSIONS

This is the first nationwide survey on incident reporting in Japan, indicating a low experience of incident reporting among physicians‐in‐training. Half of the junior residents in this study had not submitted an incident report during the previous year. Reasons for the lack of reporting experience among residents in this study may include the fact that incident reporting is based on a voluntary system or that incidents may not have been recognized and thus, not reported. Previous studies showed that 94% of physicians, including residents, were involved in all incidents and adverse events.^7^ It was also reported that over half of the residents in the United States treated patients with medically induced adverse events.^8^ These results suggest that there is a high probability that incidents involving Japanese residents are not being reported nationally.

The pattern of under‐reporting suggests two important points. First, residents are potentially involved in incidents or accidents without recognizing them. The previous study in United States comparing physician's reports and medical record reviews found a similar number of events identified by both methods; however, the degree of overlap was found to be low,^9^ suggesting that residents had been involved in more events than those reported by them. Considering that only 56% of the residents in this study knew how to report an incident, they might not report even if they wanted to. Second, patient safety lectures may not have led to an increase in incident reporting since 96% of the residents had attended lectures on patient safety. Although the content of the lectures was not investigated in this study, it is possible that the lectures did not lead to increased resident reporting behavior. These results indicate that team training practices to rehearse the techniques recommended in the team‐building model and the feedback received might be alternative methods to enhance reporting culture.^10^

While an increase in incident reporting does not necessarily guarantee that the hospital's reporting system will be effective, the reporting system may not be sufficiently effective without resident reporting. Therefore, rather than simply implementing an incident reporting system, hospitals and physicians should develop implementation strategies to increase incident reporting. Further, resident incident reports should continue to be measured, as they are an important management tool to identify factors contributing to adverse events involving residents.

This study had several limitations. First, this study was based on a questionnaire survey and did not measure actual report submission behavior or direct knowledge about the reporting system. Second, not all residents in the country were surveyed. Although hospital and individual characteristics should be considered, 38% of all Japanese physicians‐in‐training in 2020 participated in this survey,^11^ making it the largest survey of incident reporting behavior to date. Third, the reporting standards and the content of safety training varied among hospitals. Since residents are influenced by the overall number of physicians reporting and their teaching system,^12^ future studies should investigate the contexts that inhibit reporting.

Incident reporting is the cornerstone of safety culture in hospitals. This foundation can be strengthened by addressing the discrepancy between attendance at safety courses and incident reporting behavior of physicians‐in‐training.

## CONFLICT OF INTEREST

None.

## ETHICAL APPROVAL

This study was approved by the institutional review board of the Mito General Hospital (number 18‐37).

## Data Availability

The data that support the findings of this study are available from the corresponding author, upon reasonable request.
